# A bibliometric analysis of vaccine research on Chikungunya virus: contributors, trends, and emerging frontiers

**DOI:** 10.3389/fimmu.2025.1692556

**Published:** 2025-12-10

**Authors:** Da Shao, Zengwei Kou

**Affiliations:** 1 Research Center of Translational Medicine, Shanghai Children’s Hospital, School of Medicine, Shanghai Jiao Tong University, Shanghai, China; 2 Department of Laboratory Medicine and Pathobiology, Temerty Faculty of Medicine, University of Toronto, Toronto, ON, Canada

**Keywords:** vaccine development, immunotherapy, *Alphavirus*, CiteSpace, bibliometric

## Abstract

Chikungunya virus (CHIKV), the causative agent of CHIKV fever, is an arthritogenic *alphavirus* belonging to the *Togaviridae* family. It is primarily transmitted by *Aedes aegypti* mosquitoes and has the potential to become a globally pandemic pathogen. Infection with CHIKV typically results in high fever and often debilitating, sometimes chronic polyarthralgia, as well as central nervous system complications. To date, only few highly effective drugs available for treating CHIKV infection or controlling its replication and transmission. Immunotherapeutic strategies and vaccine development are considered promising approaches, particularly nucleic acid-based vaccines, which offer the advantages of rapid development, high efficacy, and precision. In this study, we performed a bibliometric analysis (the literature search was conducted up to June 2025) using data from three major medical databases to explore research trends, geographic and institutional distribution, and key research hotspots in the field of CHIKV immunotherapy and vaccine development. Our findings reveal a rapidly growing body of literature with significant contributions from leading research institutions in the United States, particularly the University of Texas system. Among the various vaccine types studied, live-attenuated vaccines and nucleic acid-based vaccines dominate the field, with the latter showing a marked increase in research attention in recent years.

## Introduction

CHIKV, a member of the genus *Alphavirus* within the *Togaviridae* family, has recently re-emerged as a global health threat. According to the World Health Organization (WHO), numerous countries and regions have reported outbreaks of chikungunya fever in 2025, including southern China (notably Guangdong Province), France and its overseas territories, the island nation of Madagascar, and South Asian countries such as India (https://www.who.int/emergencies/disease-outbreak-news/item/2025-DON581). These widespread reports have raised concerns that CHIKV could become a pandemic pathogen with significant global impact.

CHIKV is estimated to cause approximately 16.9 million infections annually, with nearly 2.8 billion people living in regions at risk of transmission (1). The disease is characterized by an abrupt onset of high fever (>39°C) lasting 2–7 days, frequently followed by chronic, debilitating polyarthralgia that affects more than 50% of patients and may persist for several months (2). Although CHIKV fever is generally non-fatal, it is estimated to be responsible for approximately 1,000 deaths per year worldwide, with infants and young children being particularly vulnerable (1).

CHIKV is primarily transmitted to humans through the bite of infected female *Aedes aegypti* and *Aedes albopictus* mosquitoes, while other mosquito species may contribute to enzootic transmission cycles in animal reservoirs. The virus was first identified during an outbreak on the Makonde Plateau of East Africa in 1952. Major epidemic activity began with a significant outbreak in 2004–2005 that originated in Kenya and spread to islands in the Indian Ocean, resulting in over half a million infections and tens of thousands of deaths. This event marked the virus’s efficient spread beyond the African continent and was followed by further global expansion (3).

Phylogenetically, CHIKV is classified into four major genotypes: West African (WA), East/Central/South African (ECSA), Asian, and Indian Ocean Lineage (IOL). Among these, the IOL genotype-distinguished by the E1-A226V mutation-has enhanced transmissibility via *Aedes albopictus*, contributing significantly to explosive outbreaks in India, Southeast Asia, and parts of Europe such as Italy and France. The Asian genotype, historically circulating in Southeast and South Asia, was introduced into the Americas around 2013, becoming the first lineage to establish local transmission in the Western Hemisphere (4).

Structurally, CHIKV consists of a ~12-kb single-stranded positive-sense RNA genome enclosed within an icosahedral capsid. The viral envelope is studded with transmembrane glycoproteins, primarily E1 and E2 (5, 6). These glycoproteins are derived from the precursor protein P62, which undergoes proteolytic cleavage by host cell furin into E2 and E3 during viral maturation. Under the acidic conditions of the host cell’s secretory pathway, E1, E2, and E3 undergo an acid-triggered conformational rearrangement into a mature spike-like trimeric structure. This spike complex plays a crucial role in viral entry by interacting with the human host cell surface receptor MXRA8 (also known as DICAM, ASP3, or Limitrin), thereby facilitating membrane fusion and subsequent internalization of the virus (6).

The structural architecture of CHIKV directly informs its antigenic properties and the immune responses it elicits. The envelope glycoproteins E1 and E2, particularly their surface-exposed domains, constitute the primary targets for neutralizing antibodies (5, 7). Studies have identified domain B of the E2 glycoprotein as the immunodominant region, responsible for triggering the majority of potently neutralizing antibodies in convalescent individuals (8). Additional neutralizing epitopes have been characterized within the E1 domain II fusion loop and the E2 domain A, which participates in human receptor binding (9). This precise understanding of CHIKV antigenicity has guided rational vaccine design, as successful immunization strategies must present these critical epitopes in their native conformational states to elicit protective antibody responses.

In response to increasing CHIKV burden, substantial efforts have been made toward vaccine development. The first live-attenuated vaccine, IXCHIQ, developed by Valneva in partnership with Bavarian Nordic, has received approval from regulatory agencies including the U.S. FDA, Health Canada, and the European Medicines Agency (EMA). However, in many countries of the world, they are not yet covered by effective vaccines and treatments. In recent years, rapid advances in nucleic acid vaccine and nanoparticle technologies-accelerated by experiences from COVID-19 vaccine development-have spurred growing interest in CHIKV immunoprophylaxis. Multiple vaccine candidates, such as virus-like particle (VLP)-based vaccines, are currently undergoing clinical evaluation (10, 11).

In this study, we employed a bibliometric approach to systematically analyze CHIKV-related literature on immunotherapy and vaccine development using data from three major biomedical databases. We identified the most productive countries, institutions, and authors in this field, and highlighted key research trends and emerging hotspots. Notably, live-attenuated vaccines remain the most frequently studied platform, while research on nucleic acid-based vaccines shows the most rapid growth. Furthermore, we proposed that artificial intelligence (AI) could be utilized in CHIKV research and present a proof-of-concept structural design of a mini-binder with potential to inhibit viral transmission, guided by AI-based protein modeling.

## Data acquisition and bibliometric analysis

To comprehensively assess research trends in CHIKV vaccines and immunotherapies, we conducted a systematic literature search (the literature search was conducted up to June 2025) in three major scientific databases: PubMed, Web of Science (WOS), and Scopus. In PubMed, the following search strategy was employed: (“Chikungunya virus”[MeSH] OR “Chikungunya fever”[MeSH] OR chikungunya) AND (vaccine OR vaccination OR immunization). For Web of Science, we used an equivalent search query: TS=(chikungunya AND (vaccine OR vaccination OR immunization)). In Scopus, the search string was defined as: TITLE-ABS-KEY (chikungunya AND (vaccine OR vaccination OR immunization)).

The search was conducted without language restrictions, and the results were exported in compatible formats for bibliometric analysis as previously described (12–14). All retrieved data were analyzed using CiteSpace and the Bibliometrix R-package for keyword co-occurrence, institutional collaboration, author productivity, and thematic mapping. Graphs and summary visualizations were further generated using GraphPad Prism and R. For statistical comparisons between two independent groups, two-tailed Student’s T-tests were performed. It is worth noting that we excluded data from 2025 when fitting the trends for annual publication volume and cumulative publication volume.

## Results

### Basic information, transmission, and global circulation of CHIKV

CHIKV has emerged as a globally significant arboviral threat (15). The geographical distribution continues to expand, with countries located between the equator and the Tropics of Cancer and Capricorn facing particularly high risk of CHIKV circulation. Recent large-scale outbreaks, including the La Réunion Island epidemic and the 2025 outbreak in Guangzhou, China, highlight the virus’s persistent epidemic potential and adaptation to new ecological niches. Current research in CHIKV basic virology primarily focuses on understanding viral evolution (16) and adaptation mechanisms (17). In epidemiology, major efforts are directed toward predictive modeling of outbreak dynamics and investigating the interplay between climate change and vector distribution expansion (18). Clinical research is increasingly concentrated on characterizing long-CHIKV syndrome and identifying biomarkers for persistent arthralgia (19), while simultaneously advancing novel therapeutic strategies and vaccine candidates (17).

**Figure 1:**
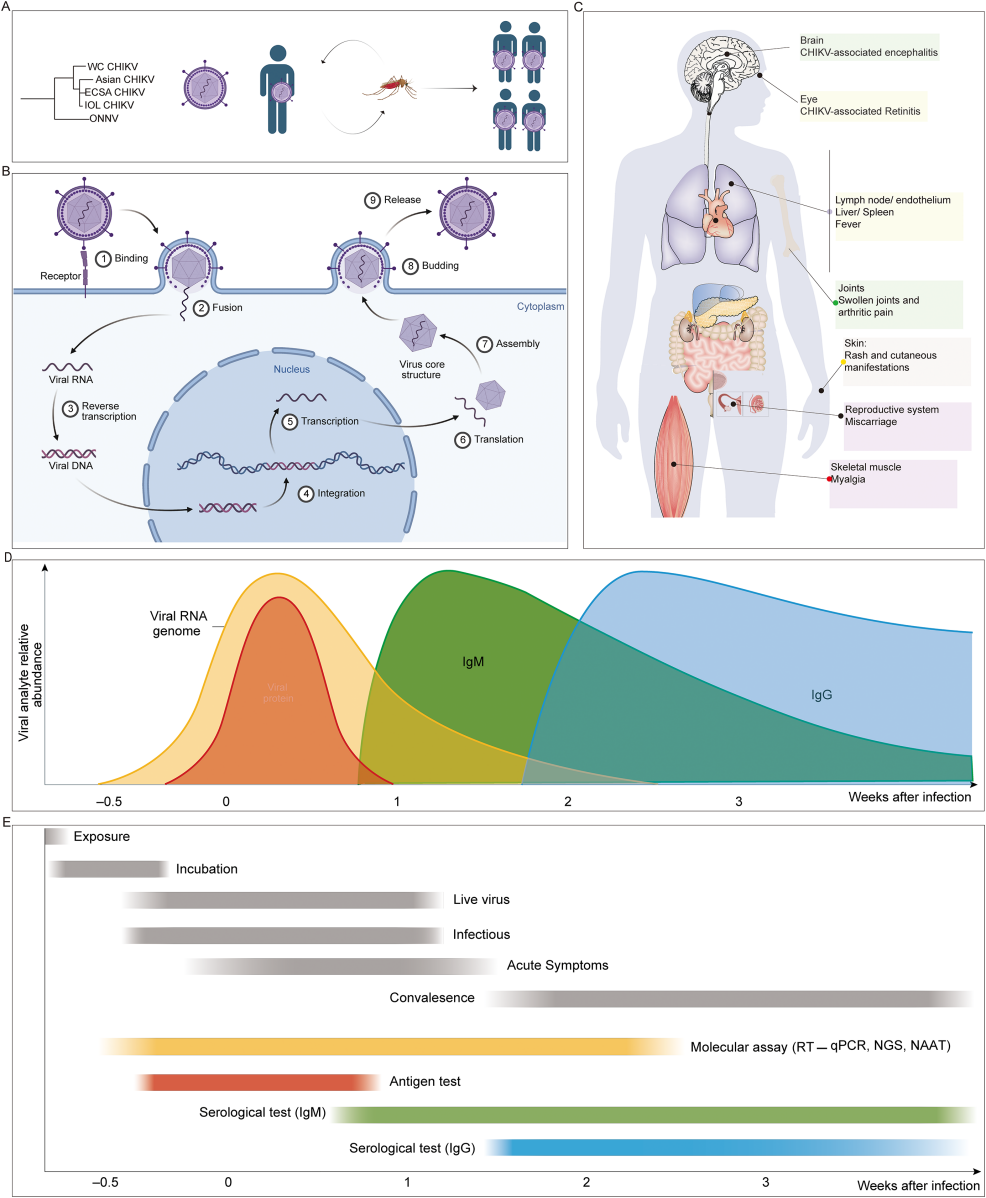
Basic information on CHIKV. **(A)** Major CHIKV lineages and transmission routes. **(B)** Viral entry via human host receptors, replication, and release of new viral particles. **(C)** Clinical symptoms caused by CHIKV infection. **(D)** Temporal dynamics of immune responses following CHIKV infection. **(E)** Diagnostic approaches for CHIKV detection.

Based on phylogenetic analyses, CHIKV has been classified into four major genotypes: West African (WC), East/Central/South African (ECSA), Indian Ocean Lineage (IOL), and Asian. The WC genotype is considered the most ancient lineage, largely restricted to West Africa. The ECSA genotype originated in Africa and represents the evolutionary source of subsequent global dissemination. The IOL genotype, which emerged from the ECSA lineage, acquired the adaptive E1-A226V mutation, markedly enhancing transmission efficiency in *Aedes albopictus*, and was responsible for the large-scale Indian Ocean outbreak in 2004-2006. In contrast, the Asian genotype arose from the ECSA lineage after long-term circulation in Asia, and has since become an independent lineage, eventually spreading to the Americas after 2013 (**Figure 1A**).

Upon human infection via mosquito bite, CHIKV enters the bloodstream, where its envelope glycoproteins engage the human host receptor MXRA8 (also known as DICAM, ASP3, or Limitrin) to mediate viral entry. Viral RNA is released into the cytoplasm of susceptible host cells, including epithelial, myeloid, and mesenchymal cells, where it serves as a template for replication and protein synthesis. Structural proteins assemble into viral particles, which are subsequently released from host cells through budding (**Figure 1B**) (20).

The incubation period of CHIKV infection generally ranges from 2 to 6 days, and the acute febrile phase typically lasts about one week. Patients are commonly present with high fever and arthralgia, while in some cases complications such as encephalitis (21), retinitis (22), and cutaneous rashes have been reported. In addition, multiple case reports indicate that maternal infection may result in miscarriage or fetal death (23). Specifically, observations from Réunion Island have revealed that maternal perinatal infection can lead to mother-to-child transmission through the placenta (24), which may cause septicemia or meningitis in infants (25). Furthermore, some children may develop cognitive and learning difficulties years later, suggesting potential involvement of the central nervous system (26). A subset of patients develops persistent joint pain, referred to as long-CHIKV, although most cases resolve within six months (**Figure 1C**).

Similar to other RNA viral infections, the immune response to CHIKV is characterized by an early IgM response, followed by the production of more durable IgG antibodies (27) (**Figure 1D**). During the acute infection stage, RT-PCR and other molecular assays allow reliable detection of viral RNA within the first two weeks. In contrast, serological detection of IgM and IgG antibodies provides diagnostic and epidemiological value in later stages. The persistence of IgG is generally associated with short-term and long-term protection against reinfection (**Figures 1D, E**) (28–31). Additionally, memory B cells and T cell-mediated immune responses contribute to sustained immunological memory, which may confer lifelong immunity against the same CHIKV lineage (32).

### Top publishing countries and institutions

**Figure 2:**
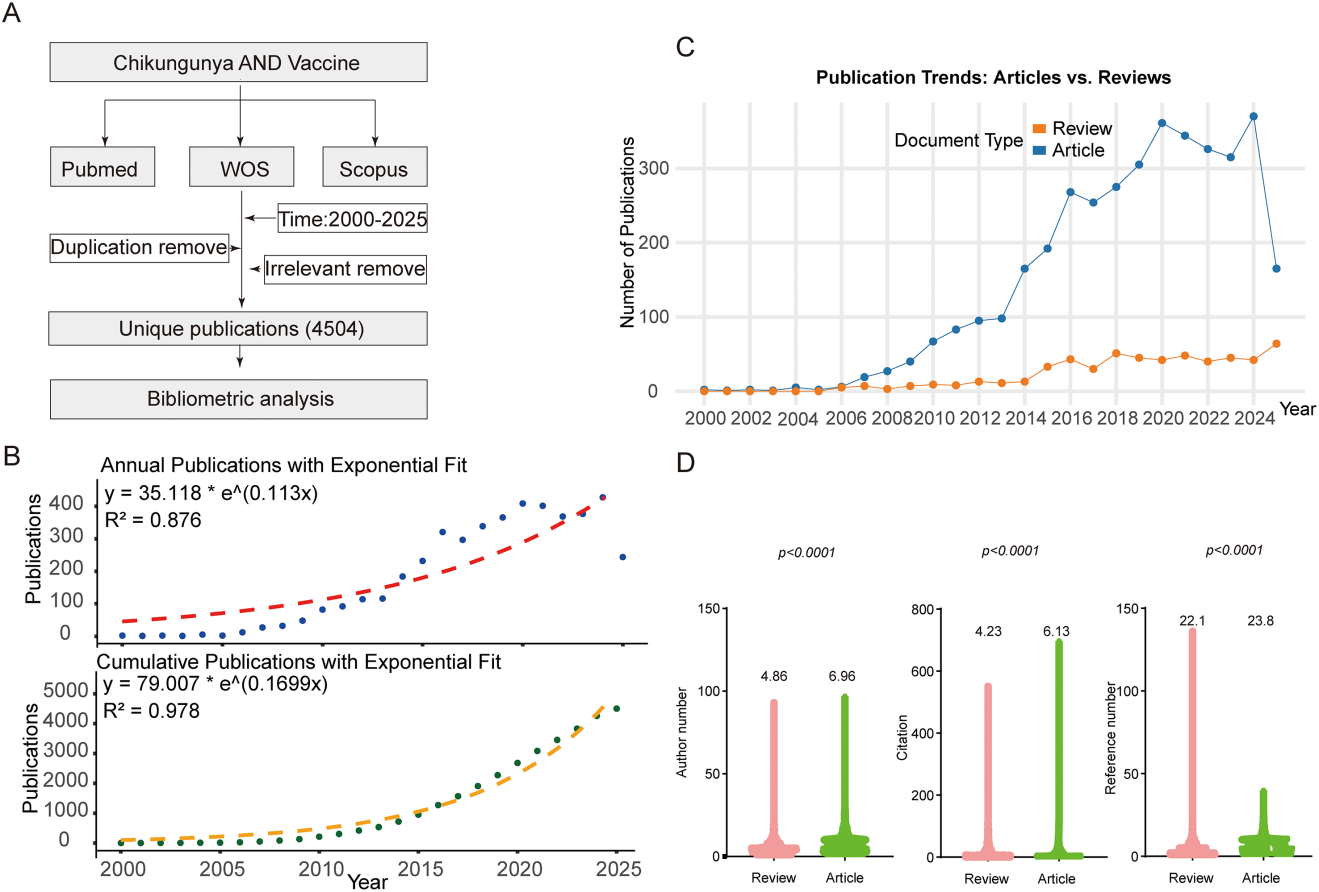
Search strategy and publication trends. **(A)** Literature retrieval strategy. **(B)** Annual and cumulative publication output over time with exponential model fitting. **(C)** Temporal trends of articles and reviews. **(D)** Comparison of articles and reviews in terms of number of authors, citations, and references.

Based on our search strategy, a total of 4,504 publications on CHIKV research from 2000 to 2025 were retrieved from PubMed, Web of Science (WOS), and Scopus (**Figure 2A**). Both the annual number of publications and cumulative publications showed rapid growth, with the annual publication trend fitting an exponential growth model (R² = 0.978) (**Figure 2B**). Regarding publication type, we found that most papers were original research articles, with a smaller proportion being reviews. Notably, by 2019 the number of articles published per year exceeded 300. Further analysis revealed that reviews had significantly higher values compared with articles in terms of author number, citation counts, and reference numbers (**Figures 2C, D**).

**Figure 3:**
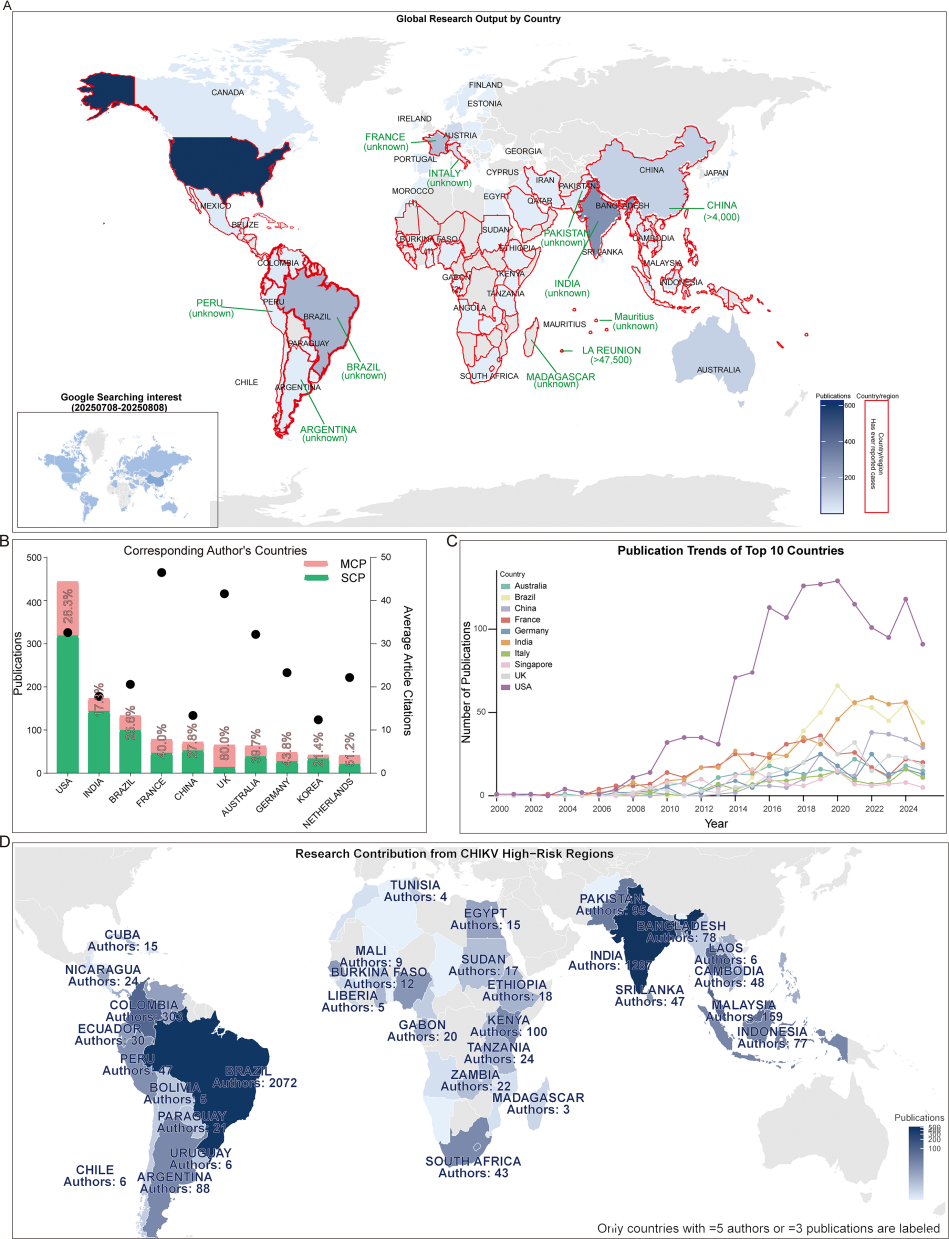
National publication trends. **(A)** World map integrating publication output by countries and countries affected by CHIKV epidemics. The inserted map shows Google search interest in CHIKV from July 8 to August 8, 2025. The blue areas represent CHIKV research output, with darker shades indicating higher publication volumes. The red areas denote countries and regions that have previously reported CHIKV outbreaks. **(B)** Top 10 corresponding-author countries and their average citations per article. **(C)** Annual publication trends of the top 10 most productive countries. **(D)** Research contribution from CHIKV high-risk regions.

By merging publication output by countries with regions previously affected by CHIKV outbreaks, we observed a striking overlap (**Figure 3A**). At the national level, the United States contributed more than 400 publications as corresponding author addressing, more than twice that of the second-ranked India. A considerable proportion of U.S. publications involved international collaboration (>28.3%) Analysis of international collaboration rates among the top 10 corresponding author countries revealed substantial variation: the United Kingdom demonstrated the highest rate of international cooperation (80.0%), followed by the Netherlands, Germany, and France, all exceeding 40%. In contrast, India, despite ranking second in total publication output, exhibited a relatively low international collaboration rate of 17.3%. Interestingly, France, ranked fourth in publication volume, achieved the highest average citation per article (46.5), nearly four times greater than that of Korea, which ranked last with an average of 12.4 citations (**Figures 3A, B**). In terms of publication trends, the United States became the leading country as early as 2009 and has remained dominant ever since (**Figure 3C**). We further analyzed research patterns in CHIKV high-risk regions, including Central and South America, Africa, the Caribbean, and South Asia, along with the contributions of authors from these areas. Our analysis revealed substantial author participation from these regions, with particularly significant research engagement from Brazil in South America, India in South Asia, and South Africa in Africa (**Figure 3D**). These three countries demonstrated the highest level of researcher involvement in CHIKV studies among all high-risk regions.

**Figure 4:**
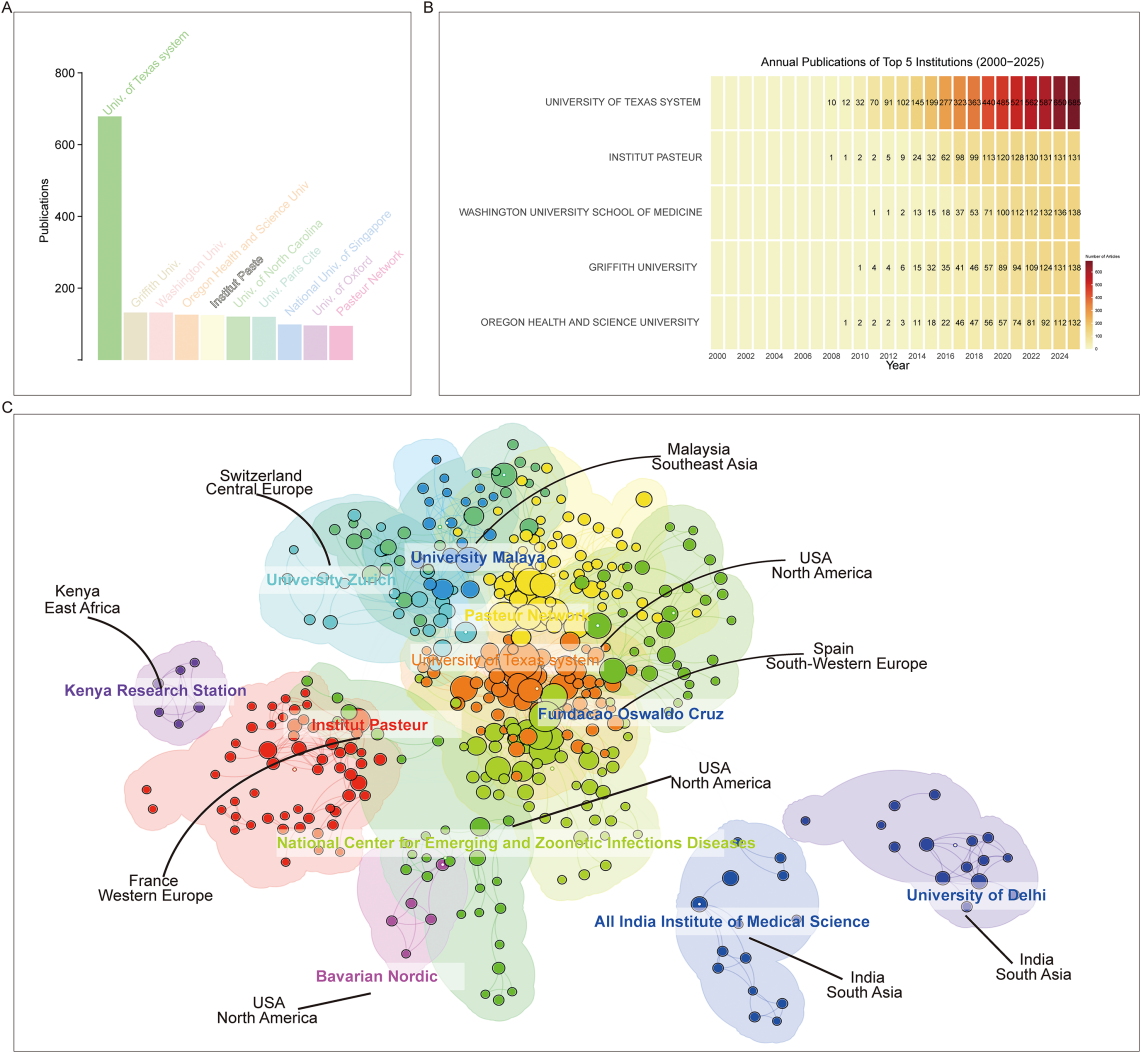
Institutional contributions. **(A)** Publications of the top 10 most productive institutions. **(B)** Annual publication trends of the top five institutions. **(C)** Institutional co-occurrence networks.

At the institutional level, among the top ten most productive affiliations, we identified three institutions associated with the University of Texas, namely the University of Texas Medical Branch, the University of Texas System, and the University of Texas Medical Branch at Galveston. These institutions (University of Texas system) consistently maintained high publication output throughout the study period, particularly after 2010 (**Figures 4A, B**). In terms of collaborative networks, the University of Texas Medical Branch at Galveston formed a large central hub, closely connected with Fundação Oswaldo Cruz and the Pasteur Network. In addition, several other institutions, including the University of Zurich, University of Malaya, and the National Center for Emerging and Zoonotic Infectious Diseases, also emerged as important hubs in the global collaboration landscape. Furthermore, the University of Delhi, the All-India Institute of Medical Sciences, and the Kenya Medical Research Institute formed their own distinct collaborative cores (**Figure 4C**).

### Journals and authors with the highest publication output

**Figure 5:**
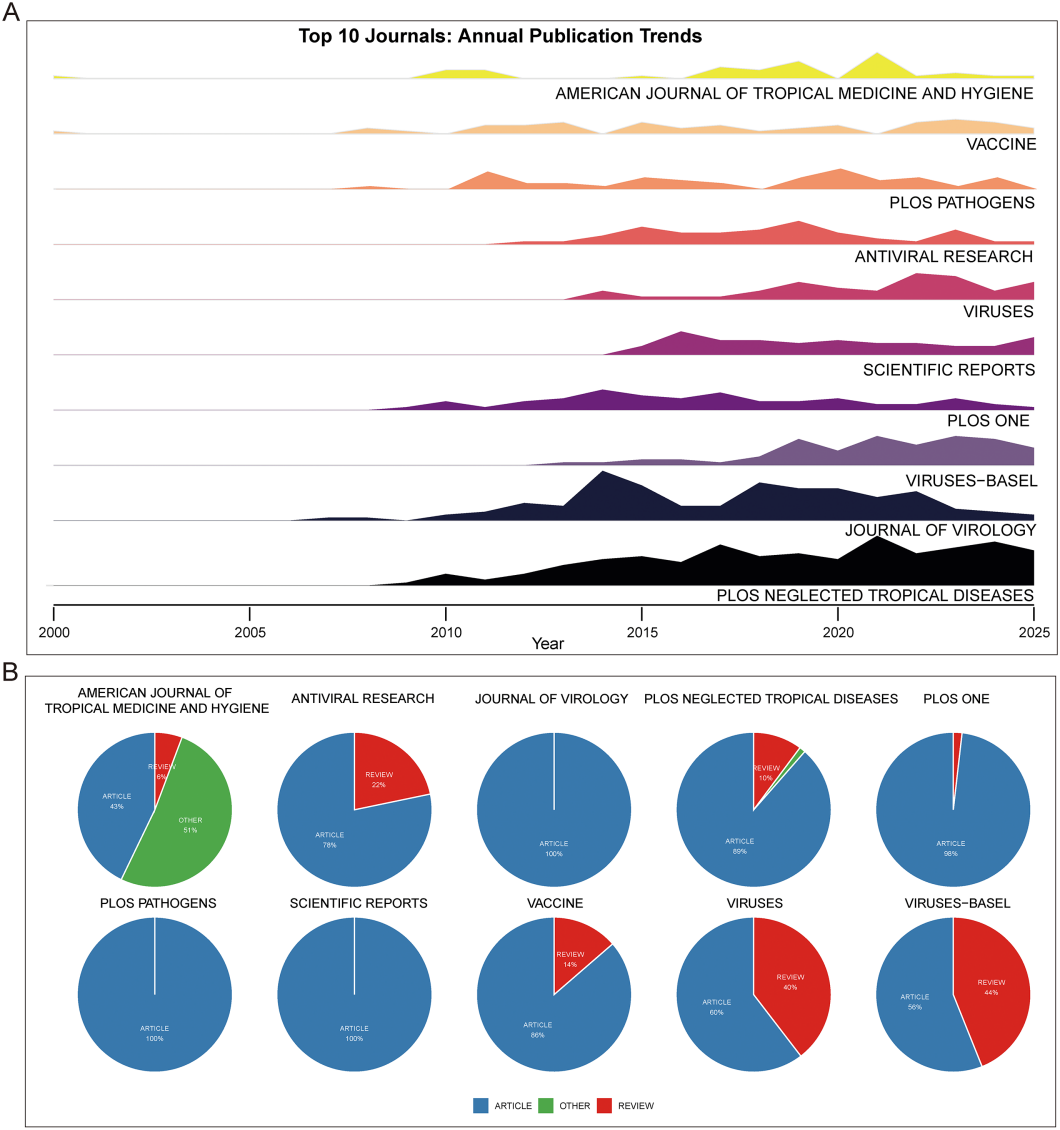
Journals and publication types. **(A)** Ridge plot showing publication trends over time in the top 10 journals. **(B)** Pie chart of publication types (articles, reviews, others) in the top 10 journals.

We observed that most of the top 10 journals with the highest publication volume began publishing extensively in this field between 2005 and 2010. Only *Virus* and *Scientific Reports* entered the field later, around 2010-2015, which may be partly attributable to their later establishment. Publication trajectories also varied among journals: for example, *PLOS Neglected Tropical Diseases* and *Viruses-Basel* showed a steady increase over time, whereas *Journal of Virology* displayed a quasi-normal distribution with a clear publication peak during the observation period. In contrast, *PLOS Pathogens* exhibited irregular fluctuations without a consistent trend (**Figure 5A**). When evaluating article types, we found that most journals predominantly published original research articles rather than reviews, with the notable exception of the *American Journal of Tropical Medicine and Hygiene*, likely due to its higher proportion of case reports, comments, and letters. Interestingly, three journals-*Journal of Virology*, *PLOS Pathogens*, and *Scientific Reports*-published almost exclusively research articles (**Figure 5B**).

**Figure 6:**
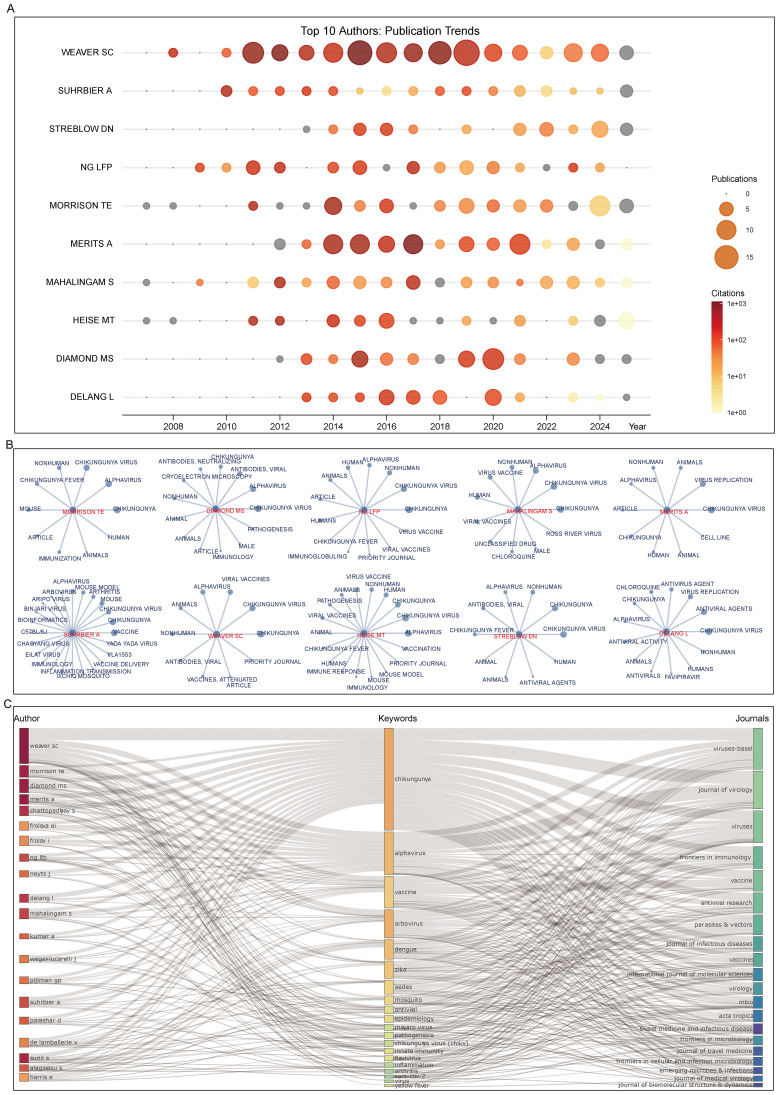
Productive authors. **(A)** Publication and citation trends of the top 10 authors. **(B)** Top keyword usage among the top 10 authors. **(C)** Three-field plot linking authors, author keywords, and journals.

Among the top 10 authors, the majority were most active between 2010 and 2020. Representative authors such as Weaver SC and Merits A reached peaks in publications and citations in 2019 and 2014, respectively. Many of the top authors have been engaged in this field for over 15 years, including Heise MT and Morrison TE (**Figure 6A**). Analysis of the top keywords among these authors revealed that at least five frequently included immunization-related terms, such as immunology, immune response, and antibody. Authors whose high-frequency keywords specifically involved vaccines included Ng LFP, Mahalingam S, Suhrbier A, Weaver SC, Heise MT, and Delang L. Several authors high-frequency keywords also pertain to antiviral agents (**Figure 6B**).

A three-field plot illustrates the relationships among authors, author keywords, and journals showed that all authors contributed to topics including Chikungunya virus, *Alphavirus*, and Chikungunya. Seven authors were involved in the Vaccine topic. High-frequency topics also encompassed other arboviruses, such as dengue, Zika virus, and *Aedes aegypti*, explaining why some authors focused on vector studies. At the journal level, *Viruses-Basel* and *Viruses* published articles covering all these topics (**Figure 6C**).

### Most influential articles and research hotspots

**Figure 7:**
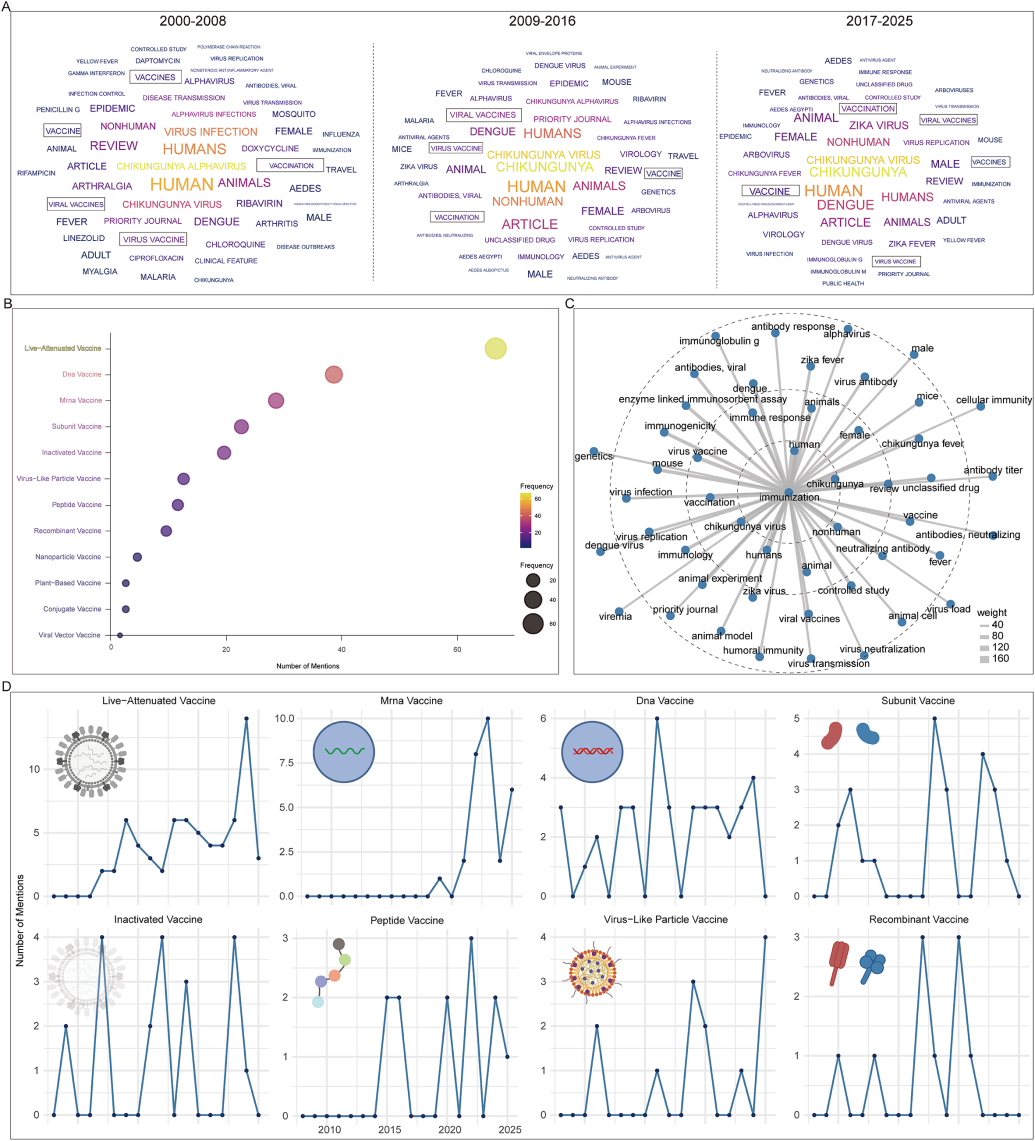
Evolution of research hotspots. **(A)** Keyword clouds across different time periods. **(B)** Frequency of different vaccine types studied. **(C)** Top 50 co-occurring terms with “immunization”. **(D)** Temporal distribution of publications on different vaccine types.

To further explore research hotspots, we conducted a word cloud analysis across three time periods: 2000-2008, 2009-2016, and 2017-2025. In all phases, vaccine-related terms (e.g., vaccine, viral vaccines, vaccination) consistently appeared among the most frequent keywords. Immune-related terms, including immunization and immunology, also maintained high prominence, together with CHIKV-associated terms such as *Alphavirus* and other mosquito-borne viruses like Zika virus. Keywords associated with antiviral strategies, such as antiviral agents, remained highly recurrent across all stages (**Figure 7A**). Analysis of vaccine-specific mentions in abstracts revealed that live-attenuated vaccines were the most frequently cited category, followed by nucleic acid vaccines, including DNA vaccines and mRNA vaccines. Subunit vaccines and inactivated vaccines were moderately represented, whereas viral vector vaccines were cited the least (**Figure 7B**).

Co-occurrence analysis showed that immunization was most strongly linked with chikungunya and human, followed by closely associated terms such as vaccination, humans, animals, immune response. Infection-related terms such as fever and virus infection also appeared, as well as post-immunization immune correlates, including immunoglobulin G, neutralizing antibody, virus neutralization, and humoral immunity. Other arboviruses, such as Zika fever and *Alphavirus*, also exhibited frequent co-occurrence. In contrast, weaker associations were observed with terms related to cellular immunity, antibody titer, neutralization, and viral load (**Figure 7C**).

### Landscape of vaccine development and the emerging role of artificial intelligence

Our analysis of vaccine platforms for CHIKV prevention identified six major vaccine types under active investigation: nucleic acid-based (DNA and mRNA vaccines), protein-based (recombinant and subunit vaccines), and virus-based (inactivated, virus-like particle, and live-attenuated vaccines). Chronological analysis revealed distinct temporal patterns among these platforms. Live-attenuated and mRNA vaccines demonstrated a general upward trend in research attention over time, with live-attenuated vaccines emerging approximately a decade earlier than mRNA platforms. In contrast, other vaccine types including DNA and subunit vaccines showed more fluctuating annual mention frequencies. By 2024, the relative research attention across vaccine types was ranked as follows: live-attenuated vaccine > inactivated vaccine = peptide vaccine > mRNA vaccine (**Figure 7D**). Analysis of the most influential references further highlighted platform preferences, with DNA vaccines (n = 2), virus-like particle vaccines (n = 3), and live-attenuated vaccines (n = 5) being the most represented categories among the top 25 references with the strongest citation bursts (**Table 1**).

**Table 1:** Top 25 References with the Strongest Citation Bursts.

ID	Title	DOI	Begin	End	Strength	Year	Type
1	A Single Mutation in Chikungunya Virus Affects Vector Specificity and Epidemic Potential	10.1371/journal.ppat.0030201	2008	2012	21.5011	2007	
2	Changing patterns of chikungunya virus: re-emergence of a zoonotic arbovirus	10.1099/vir.0.82858-0	2008	2012	19.7949	2007	
3	Infection with chikungunya virus in Italy: an outbreak in a temperate region	10.1016/S0140-6736(07)61779-6	2008	2012	19.7949	2007	
4	Genome Microevolution of Chikungunya Viruses Causing the Indian Ocean Outbreak	10.1371/journal.pmed.0030263	2008	2011	19.4098	2006	
5	A Mouse Model for Chikungunya: Young Age and Inefficient Type-I Interferon Signaling Are Risk Factors for Severe Disease	10.1371/journal.ppat.0040029	2009	2013	20.4567	2008	
6	Immunogenicity of novel consensus-based DNA vaccines against Chikungunya virus	10.1016/j.vaccine.2008.03.060	2009	2013	18.2926	2008	DNA vaccine
7	A virus-like particle vaccine for epidemic Chikungunya virus protects nonhuman primates against infection	10.1038/nm.2105	2010	2015	31.0373	2010	Virus-like vaccine
8	Chikungunya disease in nonhuman primates involves long-term viral persistence in macrophages	10.1172/JCI40104	2010	2015	21.2893	2010	live-attenuated vaccine
9	Chimeric alphavirus vaccine candidates for chikungunya	10.1016/j.vaccine.2008.07.054	2010	2013	19.7967	2008	Virus-like vaccine
10	Prophylaxis and Therapy for Chikungunya Virus Infection	10.1086/600381	2010	2014	18.4071	2009	
11	Glycoprotein organization of Chikungunya virus particles revealed by X-ray crystallography	10.1038/nature09555	2011	2015	21.9791	2010	
12	Chikungunya Virus Arthritis in Adult Wild-Type Mice	10.1128/JVI.02603-09	2011	2015	19.7147	2010	
13	Biology and pathogenesis of chikungunya virus	10.1038/nrmicro2368	2011	2015	18.9431	2010	
14	A DNA Vaccine against Chikungunya Virus Is Protective in Mice and Induces Neutralizing Antibodies in Mice and Nonhuman Primates	10.1371/journal.pntd.0000928	2011	2016	18.2912	2011	DNA vaccine
15	Novel Chikungunya Vaccine Candidate with an IRES-Based Attenuation and Host Range Alteration Mechanism	10.1371/journal.ppat.1002142	2012	2016	17.9288	2011	live-attenuated vaccine
16	Chikungunya virus and prospects for a vaccine	10.1586/erv.12.84	2013	2017	23.4514	2012	
17	Safety and tolerability of chikungunya virus-like particle vaccine in healthy adults: a phase 1 dose-escalation trial	10.1016/S0140-6736(14)61185-5	2015	2019	20.8722	2014	Virus-like vaccine
18	Chikungunya Virus and the Global Spread of a Mosquito-Borne Disease	10.1056/NEJMra1406035	2016	2020	20.2701	2015	
19	Vaccine and Therapeutic Options To Control Chikungunya Virus	10.1128/CMR.00104-16	2018	2023	17.7014	2018	
20	Chikungunya virus: epidemiology, replication, disease mechanisms, and prospective intervention strategies	10.1172/JCI84417	2019	2022	18.8974	2017	
21	Mxra8 is a receptor for multiple arthritogenic alphaviruses	10.1038/s41586-018-0121-3	2019	2023	18.691	2018	
22	Immunogenicity, safety, and tolerability of the measles-vectored chikungunya virus vaccine MV-CHIK: a double-blind, randomised, placebo-controlled and active-controlled phase 2 trial	10.1016/S0140-6736(18)32488-7	2019	2023	17.9229	2018	live-attenuated vaccine
23	Single-shot live-attenuated chikungunya vaccine in healthy adults: a phase 1, randomised controlled trial	10.1016/S1473-3099(20)30238-3	2021	2025	21.5681	2020	live-attenuated vaccine
24	The global epidemiology of chikungunya from 1999 to 2020: A systematic literature review to inform the development and introduction of vaccines	10.1371/journal.pntd.0010069	2022	2025	17.707	2022	
25	Effectiveness of CHIKV vaccine VLA1553 demonstrated by passive transfer of human sera	10.1172/jci.insight.160173	2023	2025	18.3545	2022	live-attenuated vaccine

**Figure 8:**
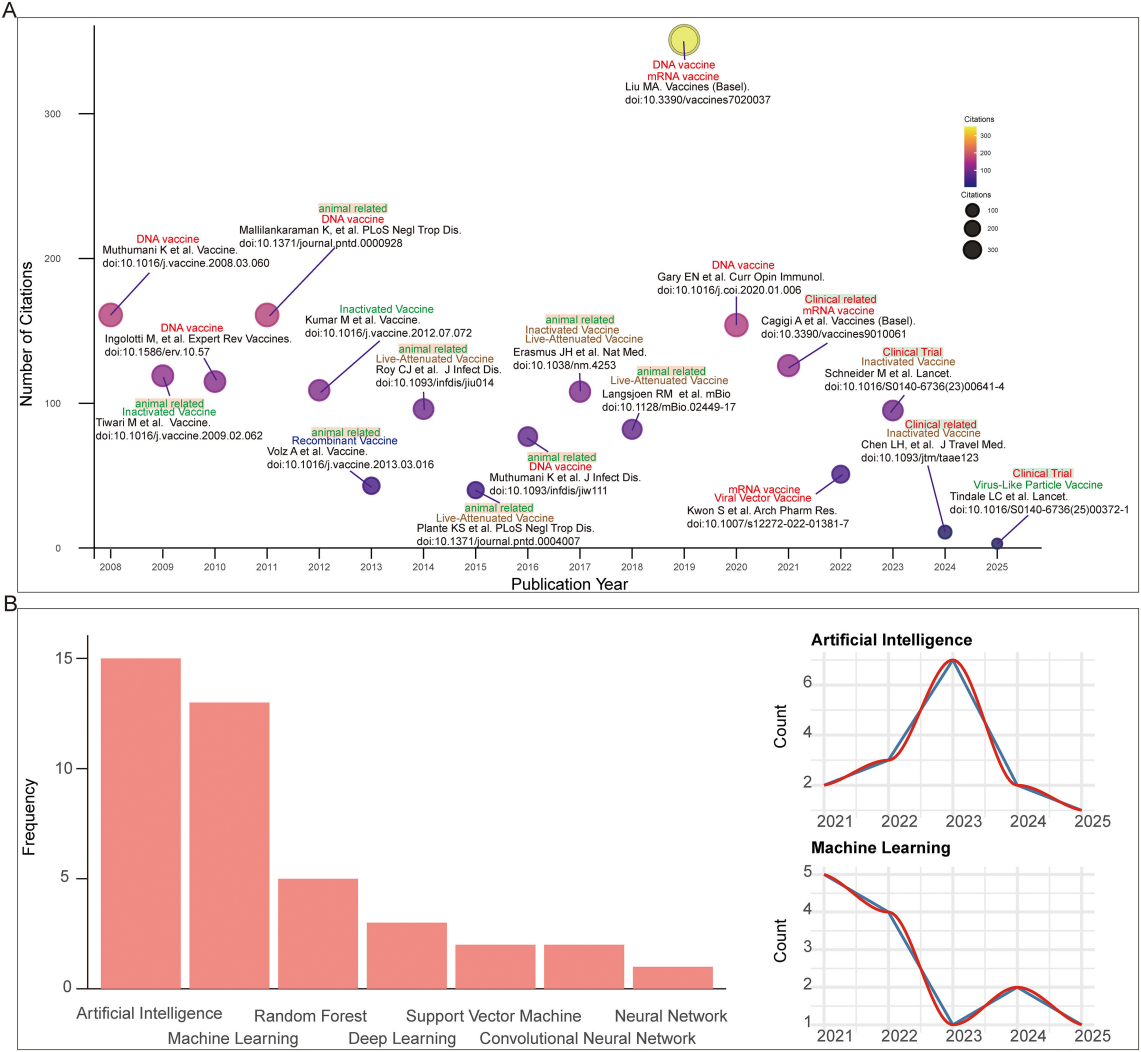
Evolution of research hotspots and AI-related research. **(A)** Plot of basic information on the most highly cited vaccine-related papers annually and the types of vaccines mentioned. **(B)** Frequency and temporal trends of studies mentioning artificial intelligence in the CHIKV field.

We further analyzed publications that explicitly mentioned “vaccine” in **Figure 7B** and identified the most highly cited paper for each year. Our analysis revealed that since 2008, eight highly cited papers involved animal studies, while four focused on clinical trials, with the latter concentrated predominantly in recent years. Consistent with our previous findings, nucleic acid-based vaccines (DNA and mRNA vaccines) and live-attenuated vaccines constituted the majority of these influential publications. Notably, the 2019 review article (doi:10.3390/vaccines7020037) by Liu MA in the journal Vaccine, which focused on nucleic acid vaccines, achieved the highest citation count (>300 citations) (**Figure 8A**).

In parallel, we observed a notable emergence of artificial intelligence (AI)-related terms within our database, with “artificial intelligence” appearing 15 times, followed by “machine learning”, “random forest”, “deep learning”, “support vector machine”, “convolutional neural network”, and “neural network” (**Figure 8**). The peak of AI-related publications occurred in 2023, during which it was mentioned in seven studies. While machine learning-related studies emerged in 2021 with five publications, their frequency declined in subsequent years. Further analysis of AI-tagged papers revealed that AI and machine learning are increasingly applied in virus research to improve outbreak prediction, surveillance, and therapeutic development. Specific applications included forecasting CHIKV and dengue epidemics, optimizing vector population modeling, assessing transmission dynamics, and accelerating vaccine discovery through epitope prediction, peptide binding analysis, and in silico degradome profiling. Collectively, these findings highlight that both diverse vaccine platforms and AI-based computational approaches are central to advancing CHIKV prevention strategies (**Table 2**).

**Table 2:** Papers that related to artificial intelligence.

Title	Journal	Year	Citations	DOI
Application of big data and artificial intelligence in epidemic surveillance and containment	Intell Med	2023	21	10.1016/j.imed.2022.10.003
Modeling dengue vector population with earth observation data and a generalized linear model	Acta Tropic	2021	13	10.1016/j.actatropica.2020.105809
Inference and Learning Methodology of Belief Rule Based Expert System to Assess Chikungunya	Appl Intell Infor	2021	10	10.1007/978-3-030-82269-9_1
Peptide arrays incubated with three collections of human sera from patients infected with mosquito-borne viruses	F1000Res	2020	8	10.12688/f1000research.20981.3
Kernel-Based Machine Learning Models for the Prediction of dengue and Chikungunya Morbidity in Colombia	CCIS	2017	6	10.1007/978-3-319-66562-7_34
Study of the Binding Pattern of HLA Class I Alleles of Indian Frequency and cTAP Binding Peptide for Chikungunya Vaccine Development	Int J Pept ResTher	2020	5	10.1007/s10989-020-10038-2
T-Cell Epitope Prediction of Chikungunya Virus	Methods Mol Biol	2016	2	10.1007/978-1-4939-3618-2_18
In silico prediction of the granzyme B degradome	BMC Genomics	2012	0	10.1186/1471-2164-12-s3-s11
Chikungunya outbreak (2015) in the Colombian Caribbean: Latent classes and gender differences in virus infection	PLoS Negl Trop Dis	2020	0	10.1371/journal.pntd.0008281
A specific and portable gene expression program underlies antigen archiving by lymphatic endothelial cells	BioRxiv	2024	0	10.1101/2024.04.01.587647
Implementation of Artificial Intelligence-Based Models with Improved Hybrid Techniques in Forecasting Possible Future Outbreaks: Which Agents and When?	Conference paper	2024	0	10.1109/ASET60340.2024.10708757
A specific gene expression program underlies antigen archiving by lymphatic endothelial cells in mammalian lymph nodes	Res Sq	2024	0	10.21203/rs.3.rs-5493746/v1
Advances in antiviral strategies targeting mosquito-borne viruses: cellular, viral, and immune-related approaches	Virol J	2025	0	10.1186/s12985-025-02622-z
i-DENV: development of QSAR based regression models for predicting inhibitors targeting non-structural (NS) proteins of dengue virus	Front Pharmacol	2025	0	10.3389/fphar.2025.1605722
A novel intelligent framework for assessing within-host transmission dynamics of Chikungunya virus using an unsupervised stochastic neural network approach	Comput Biol Chem	2025	0	10.1016/j.compbiolchem.2025.108380

Collectively, our results paint a comprehensive landscape of the CHIKV research ecosystem. This field is characterized by global collaboration led by the United States and endemic nations, sustained contributions from long-term dedicated authors and institutions, and a dynamic scientific focus that has progressively intensified around diverse vaccine platforms. Looking ahead, three convergent fronts present both the greatest challenges and the most promising opportunities. First, bridging the gap between promising vaccine candidates in development and their equitable deployment in affected regions remains a paramount challenge. Second, a deeper mechanistic understanding of long-CHIKV syndrome is urgently needed to address the chronic burden of disease. Finally, the integration of emerging technologies, notably artificial intelligence for predictive modeling and antigen design, with traditional virological and clinical expertise is poised to redefine the research pace. Tackling these priorities will necessitate a new paradigm of cooperation, opening the door for structured partnerships between virologists and clinicians in endemic areas, who provide critical biological insights and clinical data, and experts in immunogen design, data science, and public health implementation from global research hubs. Uniting these complementary strengths is the key to translating scientific progress into tangible global health impacts against CHIKV.

## Discussion

In summary, our bibliometric analysis reveals that CHIKV vaccine research is a globally collaborative endeavor, led by endemic countries and major research institutions, which has undergone dynamic growth over the past two decades. Among vaccine platforms, live-attenuated vaccines remain a major focus, but nucleic acid vaccines, particularly DNA and mRNA, are rapidly emerging due to their adaptability and translational potential. Meanwhile, AI is beginning to reshape the research landscape, with applications ranging from epidemiological modeling to computational vaccine design. Together, these findings paint a picture of a field driven by both global public health needs and technological innovation, with vaccine platform diversification and AI integration standing out as the defining trends for the future of CHIKV prevention.

Research on CHIKV vaccines has attracted continuous attention over the past two decades. Annual publication output grew from only a handful of articles in the early 2000s to over 100 papers in 2012, followed by a rapid increase in subsequent years. This growth trend is well captured by the cumulative publication curve, which fits an exponential model with high accuracy (R² = 0.98, **Figure 2B**). Most CHIKV vaccine studies have been published as original articles (**Figure 2C**), a pattern also reflected in the top 10 journals. With the exception of *Viruses* and *Viruses-Basel*, where review papers outnumbered articles, original research dominated publication formats (**Figure 5B**). Geographical analysis revealed strong overlaps between the most prolific countries and those heavily affected by CHIKV outbreaks, particularly in South America, South Asia, and Africa (**Figure 3A**). This alignment underscores the link between local disease burden and research investment (1). Interestingly, no significant correlation was observed between national publication output and average citations per article (**Figure 3B**). The United States, Brazil, and India led in total publications (**Figures 3B, C**), with U.S. institutions-particularly the University of Texas system and its medical branches-acting as central hubs of collaboration. France’s Institut Pasteur also demonstrated remarkable influence, while institutions in low- and middle-income countries, such as the All-India Institute of Medical Sciences, Kenya Research Station, and University of Malaya, made substantial contributions (**Figure 4**). Collectively, these patterns emphasize the global and collaborative nature of CHIKV research, aligning with the principle of science serving human health.

At the author level, several researchers, such as Morrison TE, Mahalingam S, and Weaver SC, have been active in this field for nearly two decades. Weaver SC, for example, has consistently contributed high-impact studies since his first publication in 2008. Analysis of the top 10 authors’ keywords revealed strong attention to CHIKV and other arboviruses, including Zika virus, Ross River virus, and *Alphavirus* more broadly. In addition to vaccine and immunology research, many of these investigators also pursued antiviral reagent development (**Figure 6**). This finding was supported by keyword co-occurrence networks and word cloud analysis, where “vaccine” and “antiviral agents” consistently appeared from 2000 to 2025, albeit with varying prominence.

The attention given to different vaccine platforms in CHIKV research has varied over time (1). Live-attenuated vaccines have attracted the greatest interest (**Figure 7**), reflecting their established development pipelines, direct mechanism of action, and proven utility in other viral diseases such as COVID-19 (33, 34). These vaccines are often the fastest to be deployed during outbreaks. However, live-attenuated vaccines also present limitations, including the induction of non-neutralizing antibodies against non-fusion regions of viral proteins and reduced efficacy against rapidly evolving viral variants (35). In contrast, nucleic acid vaccines (DNA and mRNA) offer greater flexibility, rapid development cycles, scalability, high adaptability, and strong safety profiles. Their theoretical ability to adapt to viral mutations by modifying only a few amino acids has made them increasingly attractive (36). This is consistent with our observation of a rapid rise in DNA and mRNA vaccine publications in recent years.

Our analysis of the literature (**Table 2**, **Figure 8B**) reveals that the application of artificial intelligence in CHIKV research is a rapidly evolving frontier, primarily driven by computational biologists, epidemiologists, and immunologists. These researchers are leveraging AI to tackle epidemic forecasting and virus transmission and dynamics challenges in the CHIKV field. The most pertinent to vaccine development, immunologists and bioinformaticians are using AI to deconstruct the immune response and accelerate antigen design. For instance, studies [#3, #6, #7, #8 of **Table 2**] utilize AI for T-cell and B-cell epitope prediction, analysis of peptide-binding patterns to HLA alleles, and in silico prediction of immune protease targets (e.g., granzyme B degradome). The future implication here is profound: these computational approaches can drastically reduce the time and cost of the initial stages of vaccine candidate screening. By pre-selecting the most promising, conserved, and broadly immunogenic epitopes in silico, we can prioritize laboratory experiments on a handful of leads rather than thousands of possibilities, paving the way for a new generation of multi-valent or universal CHIKV vaccines.

In parallel, the introduction and maturation of *de novo* protein design technologies have enabled the rational design of functional proteins such as minibinders, which hold promise for CHIKV therapeutic development. While our bibliometric tracking suggests that AI applications in CHIKV research have so far been concentrated on outbreak prediction, vector population modeling, transmission dynamics assessment, and computational approaches such as epitope prediction, peptide binding analysis, *de novo* design of minibinders has not yet been reported in this field (**Figure 8**, **Table 2**). Nevertheless, given that structural information for CHIKV fusion proteins and their human receptor MXRA8 has already been resolved (5, 6), it is reasonable to anticipate the emergence of AI-assisted minibinder design in the near future.

In summary, our findings demonstrate that CHIKV vaccine research has evolved through dynamic growth, driven by both global disease burden and international collaboration. Among vaccine platforms, live-attenuated vaccines remain a major focus, but nucleic acid vaccines, particularly DNA and mRNA, are rapidly emerging due to their adaptability and translational potential. Meanwhile, AI is beginning to reshape the landscape of CHIKV research, with applications ranging from epidemiological modeling to computational vaccine design. Together, these advances highlight the dual drivers of vaccine innovation and AI integration as critical forces shaping the future of CHIKV prevention and control.

## Data Availability

The raw data supporting the conclusions of this article will be made available by the authors, without undue reservation.
